# 1114. Case Study. Resuscitation and Surgical Management of a Significant Burn in the Setting of Severe Aortic Stenosis

**DOI:** 10.1093/jbcr/irag033.288

**Published:** 2026-04-06

**Authors:** Syed F Saquib, Shray Malik, Pranav Patel, Kyle Digrande

**Affiliations:** University of California Irvine, Orange, CA; UCI Health, Orange, CA; UCI Health, Orange, CA; UCI Health, Orange, CA

## Abstract

**Patient Presentation (age range, injury details, relevant history):**

65 year old male who sustained flame burn total body surface area (TBSA) 15% to his back, head, back, bilateral buttocks, and bilateral upper extremities after being pushed into a firepit. Initially estimated to be 20% TBSA, Parkland Resuscitation was started. He had evidence of hypovolemia requiring fluid resuscitation via nurse driven fluid resuscitation protocol. Despite being just under 20% TBSA burn, the patient required substantial fluid resuscitation. His ECHO demonstrated paradoxical, low flow, low gradient, severe aortic valve stenosis (AS) and acute diastolic heart failure with a preserved ejection fraction (65%).

**Clinical Challenges:**

1. How to resuscitate a patient in the setting of severe AS?

2. How to optimize the patient's AS prior to surgery?

3. What's the optimal surgical excision and temporizing substitute to use?

**Management Approach:**

1.Assuming a 20% TBSA, theoretical fluid requirements over the course of the 1st 24 hours would have been 4800 cc yet he required 10 860 cc. Given consistently low urine output, 5% albumin was started about 12 hours into his resuscitation to avoid fluid creep. On hospital day (HD) 2 his hemodynamics stabilized.

2. Cardiology was asked to optimize him for surgery for his 3rd degree burns of his back and buttocks. An interdisciplinary discussion was held and the decision to perform any invasive intervention (SAVR, TAVR or valvuloplasty) was deferred until burns were excised due to the risk of infection & endocarditis.

3. On HD3, the patient was taken to the OR where a fascial excision was performed with application of biodegradable temporizing matrix with the following rationale: a. Excising to the most vascular wound from the start as he may not tolerate multiple surgeries in a brief period. b. Applying a dermal substitute that can remain in place for weeks while allowing him to recover. Postoperatively, he was placed on the ventilator and after diuresis he was liberated off the ventilator on HD#7.

**Outcomes:**

On HD#37, in preparation for autograft surgery, patient underwent cardiac catheterization with valvulopasty with a successful reduction of peak gradient by 20 mmHg and substantial improvement in his dyspnea. On HD#39, patient had placement of autograft and autologous skin cell suspension to the back and buttocks burn which achieved about 80% closure. He required 3 patch graft surgeries on the lower buttocks burn wounds to facilitate near 90% closure. Per patient request, additional surgery was deferred. He was discharged on HD#85 with cardiology and cardiac surgery follow up for definitive aortic valve replacement.

**Lessons Learned:**

Patients with substantial burns in the setting of severe AS can require large volume fluid resuscitation due to their preload dependency. Successful surgical care warrants a thoughtful interdisciplinary collaboration between Burn, Anesthesia and Cardiology to mitigate any perioperative complications while balancing the need for expeditious burn wound management.

**Applicability to Practice:**

Patient with severe AS needing multiple burn surgeries can have a successful outcome of facilitating wound closure while also addressing the patient's cardiac needs.

